# Open-source dataset reveals relationship between walking bout duration and fall risk classification performance in persons with multiple sclerosis

**DOI:** 10.1371/journal.pdig.0000120

**Published:** 2022-10-18

**Authors:** Brett M. Meyer, Lindsey J. Tulipani, Reed D. Gurchiek, Dakota A. Allen, Andrew J. Solomon, Nick Cheney, Ryan S. McGinnis

**Affiliations:** 1 Department of Electrical and Biomedical Engineering, University of Vermont, Burlington, Vermont, United States of America; 2 Department of Biomedical Engineering, University of Massachusetts Lowell, Lowell, Massachusetts, United States of America; 3 Department of Bioengineering, Stanford University, Stanford, California, United States of America; 4 Department of Neurological Sciences, Larner College of Medicine at the University of Vermont, Burlington, Vermont, United States of America; 5 Department of Computer Science, University of Vermont, Burlington, Vermont, United States of America; Tsinghua University, CHINA

## Abstract

Falls are frequent and associated with morbidity in persons with multiple sclerosis (PwMS). Symptoms of MS fluctuate, and standard biannual clinical visits cannot capture these fluctuations. Remote monitoring techniques that leverage wearable sensors have recently emerged as an approach sensitive to disease variability. Previous research has shown that fall risk can be identified from walking data collected by wearable sensors in controlled laboratory conditions however this data may not be generalizable to variable home environments. To investigate fall risk and daily activity performance from remote data, we introduce a new open-source dataset featuring data collected from 38 PwMS, 21 of whom are identified as fallers and 17 as non-fallers based on their six-month fall history. This dataset contains inertial-measurement-unit data from eleven body locations collected in the laboratory, patient-reported surveys and neurological assessments, and two days of free-living sensor data from the chest and right thigh. Six-month (n = 28) and one-year repeat assessment (n = 15) data are also available for some patients. To demonstrate the utility of these data, we explore the use of free-living walking bouts for characterizing fall risk in PwMS, compare these data to those collected in controlled environments, and examine the impact of bout duration on gait parameters and fall risk estimates. Both gait parameters and fall risk classification performance were found to change with bout duration. Deep learning models outperformed feature-based models using home data; the best performance was observed with all bouts for deep-learning and short bouts for feature-based models when evaluating performance on individual bouts. Overall, short duration free-living walking bouts were found to be the least similar to laboratory walking, longer duration free-living walking bouts provided more significant differences between fallers and non-fallers, and an aggregation of all free-living walking bouts yields the best performance in fall risk classification.

## Author summary

Falls are both highly prevalent and injurious in persons with Multiple Sclerosis (PwMS), thus we are interested in finding methods to understand the fall risk of PwMS. To examine the differences between PwMS in a clinic environment and at home, we collected and made publicly available a dataset where PwMS performed daily life activities in the clinic and then wore wearable sensors at home for two days. We found people walk very differently at home *vs* in-clinic. However, the longer they walk for, the closer their walking attributes relate to how they walk in-clinic. Additionally, in examining multiple approaches, we found both the full length and short bouts of at-home walking periods can identify the fall risk of PwMS- each providing varying levels of performance. Crucially, we find that methods and assessments developed for in-clinic methods may need to be adjusted to function properly at home and when performing walking analysis at home, analyzing differing durations of walking will impact the results.

## Introduction

Multiple Sclerosis is characterized by progressive demyelination and axonal damage throughout the central nervous system [1,2]. As a result, persons with multiple sclerosis (PwMS) experience symptoms including debilitating fatigue and impaired coordination, muscle strength, and sensation, leading to difficulty with postural control in dynamic activities which, in turn, leads to falls [3]. Over 50% of falls result in injury and 66% of first-time falls require a visit to the emergency department, reducing quality of life and yielding an estimated annual healthcare cost of $80 billion in the United States alone [4]. Of the 2.3 million PwMS globally, over half will experience a fall in any three-month period [5]. As MS is a chronic condition, injurious falls pose a substantial and long-term burden to patient quality of life and the healthcare system [6].

Given these impacts, effective fall prevention is critical. Fall risk in PwMS is difficult to assess as it is known to vary both within and across days. Fall risk may be elevated in the absence of an assistive device (e.g., walking sticks) [7] or during balance-challenging tasks, such as walking, position transfers, and changes of direction [8]. However, current clinical assessments often only occur once every six months; an observation frequency incapable of capturing the true time-varying nature of symptoms in MS, limiting the ability to prescribe preventative interventions [9]. There is a clear need for novel assessments that are sensitive to this inherent variability and that can capture the relationship between symptom fluctuations and fall risk. One approach is for assessments to incorporate continuous monitoring in free-living conditions, which provide far more than a twice-per-year snapshot of symptoms, and advanced machine learning techniques that can effectively capture the complex relationship between these movement data and fall risk.

With the growing availability of wearable sensor data, it may now be possible to leverage machine learning, and particularly *deep learning* models, to learn high-level outcomes like fall risk directly from raw sensor data without manual feature engineering [10,11]. Studies employing deep learning for time series classification tasks, such as our prior work classifying fall risk in PwMS from in-lab measurements [12] and work from others to detect falls and classify fall risk in non-MS populations with balance and mobility impairment [13–21], have found superior results when compared to machine learning techniques that rely on manually-constructed features. Notably, these results are achieved despite the significant amounts of data needed for training deep learning models. It is possible that given larger available datasets, performance of these models could improve further, but the accumulation of these large datasets remains a barrier to entry for many into the use of deep learning models for characterizing fall risk.

Remote gait monitoring in PwMS may enable continuous fall risk assessment and the deployment of personalized fall prevention interventions. In this approach, data from individual walking bouts could inform fall risk status instantaneously. This vision has motivated the development of fall risk classification models that require only wearable sensor data from a single gait bout as model inputs [12,22,23]. However, deploying these models remotely comes with additional challenges that may impact model performance. For example, it is well established in PwMS [24–26] and other populations [27–29] that gait observed in the clinic differs from gait observed remotely (especially for gait speed-dependent variables). Similarly, studies in older adults [30] and PwMS [24] have also discovered that gait parameters change with walking bout duration. However, it is currently unclear how walking bout duration relates to fall risk in PwMS [7,30], and this has not been evaluated in previous development of fall risk classification models [12,22,23].

The primary objective of this work is to share a new, open-source dataset that can help other research groups develop digital biomarkers of impairment and fall risk in PwMS. In service to this objective, we present a framework for remote gait analysis on this dataset and use it to examine how gait parameters and fall risk classification performance, based on feature-based machine learning and stride acceleration based deep learning methods, change in relation to walking bout duration in PwMS.

## Materials and methods

### Dataset: Subjects and protocol

A sample of 38 PwMS (21:17 fallers:non-fallers; 12:27 Male:Female, mean ± standard deviation age 51 ± 12 y/o), recruited from the Multiple Sclerosis Center at University of Vermont Medical Center participated in this study (exclusion: no major health conditions other than MS, no acute exacerbations within the previous three-months, ambulatory without the use of assistive devices). PwMS who self-reported to have fallen within the previous six-months were characterized as fallers based on the criteria “consider a fall as an event where you unintentionally came to rest on the ground or a lower level.” All participants were asked to return for two additional identical study visits six-months and one-year following their initial visit. Of the 38 original cohort, 28 returned for a six-month follow-up (15:13 fallers:non-fallers; 8:20 Male:Female), and 15 returned for a one-year follow-up (6:9 fallers:non-fallers;6:9 Male:Female). Patients completed self-reported 6-month fall history each visit, allowing their fall status to change at subsequent visits. The high attrition rate observed in this study was largely due to the COVID-19 pandemic, as 3 six-month and 11 one-year follow-ups were cancelled for this reason.

On the day of testing, subjects provided written informed consent to participate in the study. A neurologist with subspecialty expertise in MS completed the Expanded Disability Status Scale (EDSS) for each subject [31]. Subjects were asked to complete a fall history survey, Activities-specific Balance Confidence Scale (ABC) [32], Modified Fatigue Impact Scale (MFIS) [33], Neurological Sleep Index (NSI) [34], and Twelve Item MS Walking Scale (MSWS) [35]. Two missing NSI entries in the clinical survey data were filled using k-nearest-neighbors (n = 3) [36]. Table 1 reports demographics of the sample.

**Table 1:** Subject demographics.

Visit	Assessment	Fallers	Non-faller
Initial	N	21	17
	Age	56.0 (9.05)	45 (12.92)
	Sex	5M:16F	7M:10F
	ABC	75.0 (18.8)	91.4 (15.5)
	EDSS	3.3 (1.4)	2.3 (1.0)
	MFIS	39.8 (17.9)	29.2 (16.7)
	MSWS	55.0 (23.3)	27.5 (11.5)
	NSI	56.6 (17.2)	46.6 (22.2)
6-month	N	15	13
	Age	55.3 (10.3)	44.2 (14.0)
	Sex	4M:11F	4M:9F
	ABC	73.8 (14.1)	90.1 (11.5)
	EDSS	3.3 (1.3)	2.0 (0.8)
	MFIS	41.0 (17.2)	25.1 (19.5)
	MSWS	46.0 (22.3)	31.3 (14.0)
	NSI	58.9 (23.3)	42.6 (23.2)
1-year	N	6	9
	Age	57.0 (9.7)	49.9 (10.8)
	Sex	4M:2F	2M:7F
	ABC	72.3 (22.0)	79.3 (17.1)
	EDSS	3.6 (1.6)	2.3 (1.1)
	MFIS	38.5 (14.4)	35.9 (18.9)
	MSWS	59.7 (24.4)	38.7 (12.3)
	NSI	43.0 (23.4)	60.6 (13.0)

Mean (standard deviation) of survey results partitioned by fall status. ABC: Activity-Specific Balance Confidence; EDSS: Expanded Disability Status Scale; MFIS: Modified Fatigue Impact Scale; MSWS: MS Walking Scale; NSI: Neurological Sleep Index; N: number of subjects in group

Subjects performed several activities in the lab completed in the following order: right and left tibialis anterior maximum voluntary contraction, timed-up-and-go (TUG) [1], timed 25-foot walk test [37], 30-second chair stand test [38], lying to standing transition, three separate two-minute standing tests: tandem standing, feet shoulder-width apart eyes open, and feet shoulder-width apart eyes close, one-minute hallway walk at a self-selected pace including one turn, 30-second normal standing, 30-second upright sitting, 30-second slouch sitting, and 30 seconds each lying on back, left side, right side, and prone. During the lab visit, subjects were instrumented with MC10 BioStamp sensors. Accelerometer (31.25 Hz, ±16G) and electromyography (1000 Hz) were collected from the right and left tibialis anterior. Accelerometer (250 Hz, ±16G) and angular rate gyroscope data (250 Hz, ±2000°/s) were collected from the chest and lower back as well as bilaterally from the anterior thighs, proximal lateral shank, and dorsal aspect of the feet. Electromyography was collected to allow the investigation of foot drop, a common cause of falls in PwMS [39]. Detailed placement information can be found in Table 2. At the conclusion of the lab visit, the participants were sent home with two MC10 BioStamp sensors for 48 hours located on the medial chest and right anterior thigh measuring acceleration (31.25 Hz ± 16G) and placed in accordance with Table 2. Data from these sensors were recorded throughout the subject’s daily life. These deidentified data are available at < https://simtk.org/projects/msense_ms_adls>. This protocol was approved by the University of Vermont’s Institutional Review Board (CHRMS 18–0285). Portions of this dataset have been used previously to support the development of approaches for characterizing fall risk from lab-based gait and from in-lab and remotely tracked thirty-second chair-stand tests [12,40,41]. In these studies, raw gait data collected in lab and deep learning models were able to adequately classify fall risk, and chair-stand-tests conducted remotely and in lab provided similar levels of fall risk classification performance.

**Table 2:** Sensor Placement.

Location	Sensing Modality 1	Sensing Modality 2	Placement Details
Medial Chest	Accel: 250 Hz, ±16G	Gyro: 250 Hz, ±2000°/s	Secured to sternum just below sternoclavicular joint.
Sacrum	Accel: 250 Hz, ±16G	Gyro: 250 Hz, ±2000°/s	Between or just above PSIS
Anterior Thigh (R/L)	Accel: 250 Hz, ±16G	Gyro: 250 Hz, ±2000°/s	Anterior aspect of thigh ~25% from knee to hip
Proximal Lateral Shank (R/L)	Accel: 250 Hz, ±16G	Gyro: 250 Hz, ±2000°/s	Secured to proximal lateral shank, ~4 fingers below fibular head
Tibialis Anterior (R/L)	Accel: 31.25 Hz, ±16G	EMG: 1000 Hz	Placed on muscle belly (widest part) of TA
Dorsal Foot (R/L)	Accel: 250 Hz, ±16G	Gyro: 250 Hz, ±2000°/s	Placed on metatarsals 2–4

R/L: sensor placed symmetrically on right and left side of body; Accel: acceleration; EMG: electromyography; Gyro: gyroscopic data (angular velocity); PSIS: posterior superior iliac spine; TA: tibialis anterior

### Remote gait analysis

**Fig 1:**
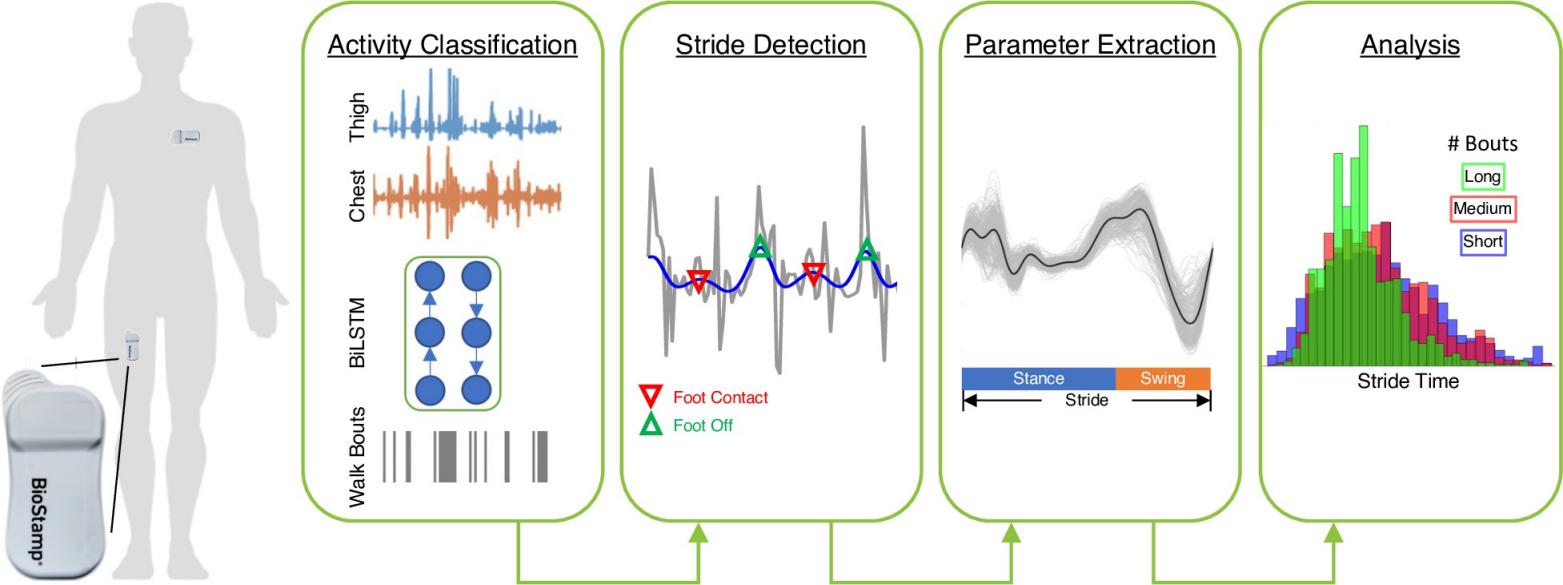
Pipeline for free-living gait analysis from BioStamp nPoint wearable sensor data. Activity classification is performed via deep neural network (BiLSTM architecture) on windows of accelerometer data sampled from the chest and thigh. Walking bouts are extracted from the resulting activity timeseries and gait events are identified using previously validated approaches to detect strides. Gait parameters are extracted from each walking bout and used for further analysis.

An overview of the remote gait analysis pipeline is presented in Fig 1. The depicted framework begins with acceleration gathered from the BioStamp sensors located on the thigh and chest followed by activity classification (e.g. finding walking), event detection within walking bouts, feature extraction, and finally analysis. Each aspect of this pipeline (gait bout identification, stride detection, parameter extraction, and analysis) are discussed in more detail below. In terms of analysis, we examine the impact of context and bout duration on discriminating fallers from non-fallers, and on the performance of feature-based and deep learning methods for classifying fall risk. These analyses are only performed on the data from the initial study visit (n = 38).

### Activity classification

Activity classification was carried out with wearable sensor data from the chest and thigh. Gait bouts were identified using a deep learning approach that leverages a Long Short Term Memory (LSTM), a type of recurrent neural network for analyzing time series data, architecture adapted from [42]. Specifically, the network is composed of a single BiLSTM layer with 215 hidden units [43], a 40% drop out layer [44], and ADAM optimization [45]. This classifier was developed using 58% data from PwMS, 26% from healthy adults, and 16% from persons with Parkinson’s Disease to provide a wide variety of example gait and non-gait data for training. Data labeled as gait were sampled from prescribed slow, comfortable, and fast walking trials completed overground, as well on a treadmill for healthy adults. Data labeled as non-gait were sampled from standing, sitting, lying, running and stair ascent and descent. Ten-fold cross validation was conducted on the training set consisting of 20,000 4-second observations (50:50 gait:non-gait) yielding validation accuracy of 98.5%. Performance on a held-out test set consisting of 3,000 observations (50:50 gait:non-gait) was 98.4%, providing evidence that the classifier is well positioned to be used on new datasets. This network was then leveraged to identify all walking bouts completed by all subjects during the 48-hour free-living wear period. Walking bouts were identified by classifying 4-second segments of data, where consecutive walking segments were concatenated into a single bout.

### Stride detection

Following walking bout identification, strides were extracted using the method described and validated in [46,47]. At a high level, this stride extraction method estimates step and stride frequency from the power spectral density of the thigh accelerometer signal. A filter bank based on these frequencies then provides the signals used to identify foot-off and foot-contact events from specific signal features. This algorithm has been validated on a wide range of walking speeds, 0.56–1.78 m/s [47], which covers the expected range of walking speeds for PwMS [48]. Bouts with fewer than two extracted strides were removed automatically before proceeding with the analysis that follows.

### Gait parameter extraction

Following walking bout and stride identification, the following features were calculated for each stride and averaged for each bout; stance time, swing time, stride time, coefficient of variation of stride time (stride time CV), duty factor, and coefficient of variation of duty factor (duty factor CV) [46]. The remaining features were calculated on the entire bout. Root mean square of the anterior-posterior acceleration from the chest sensor (RMS AP) [49], medial-lateral frequency dispersion of the chest sensor (Freqd ML) [49], and the entropy ratio between the thigh and chest [50]. Lyapunov exponent of the medial lateral (Ly ML) and anterior-posterior (Ly AP) chest sensor were calculated for gait bouts longer than 60 seconds [49].

The features mentioned above were selected based on previous literature that demonstrates their association with MS-induced gait impairment and fall risk. Stance time, swing time, and stride time have been shown to be significantly correlated with patient reported walking impairment in PwMS [51]. Stride time, duty factor [52], RMS AP, and Freqd ML have been shown to identify differences in walking impairment between PwMS and healthy controls [49]. Stride time CV has been shown to be strongly associated with fall risk in PwMS [53]. Non-linear measures, entropy ratio [50] and Lyapunov exponent in the ML and AP directions of chest acceleration [49], have been shown to capture gait stability in PwMS.

### Walking context and bout duration analysis

Gait parameter data were grouped into one of three categories based on the duration of the walking bout from which they were extracted: short—8 seconds or shorter; medium—12–28 seconds; or long—32 seconds or longer. These durations were based on results reported in other examinations of free-living gait [54]. Comparisons to gait parameters derived from lab-collected hallway-walking data and combined home data, grouped as all, were also made. Bouts where strides could not be identified or with physiologically impossible values were deleted (496 removed in total). Gait parameters for each walking bout in each duration were summarized using mean, median, max, min, standard deviation, 5^th^ percentile, and 95^th^ percentile for each subject.

Group differences in each of the gait parameters were identified using Wilcoxon Rank Sum tests between bout durations between fallers and non-fallers at each bout duration and between in-lab and free-living contexts. A significance threshold of *α* = 0.05 was used for all statistical testing.

### Feature-based fall risk classification

Statistical models that require extracted features for discriminating between individuals at high and low risk for falls were trained and tested on five different feature-sets: gait parameters calculated on short, medium, and long gait bouts, all free-living gait bouts, and in-lab gait data. These feature-sets contained one entry per identified valid walking bout. Classifier performance was established using leave-one-subject-out cross validation (LOSO-CV). In this approach, data from all but one participant (N = 37) were partitioned into a training dataset while data from the remaining subject was used for testing. This process was repeated until data from each subject had been included in the test set. The LOSO-CV approach ensures the model was tested on subjects it had not previously seen, which provides a realistic estimate of how the model would perform during real-world use. The normalized posterior probabilities, known as the decision scores, assigned to the held-out subject were combined to calculate an overall model performance by considering the area under the receiver operating characteristic curve (AUC). AUC was chosen as the main performance metric because it provides a comprehensive measure of how well a classifier is able to discriminate between groups and allows the results to be compared to other studies.

Features were normalized using z-scores then reduced using principal components analysis (PCA) within each iteration of the LOSO-CV. Prior to feature reduction, short, medium, and all-bouts have 8 features per input, long bouts have 9 features per input, and lab bouts have 11 features per input. To explain the discrepancy in the number of features, note that Entropy Ratio is computed for the long bouts and Entropy Ratio, Lyapunov Exponent AP-direction, and Lyapunov Exponent ML-direction are computed for lab walking. The principal components that explained 95% of the variance of these reduced feature sets were extracted, resulting in approximately 6 principal components for each home walking duration and 7 principal components for lab data. The reduced feature sets were then used to train Logistic Regression (LR) [55], Support Vector Machine (SVM) [56], Decision Tree [57], K-Nearest Neighbors (KNN) [58], and Ensemble of Trees (ENS) [57] binary statistical classification models to discriminate between subjects at high and low fall risk. A variety of model types were used to capture different relationships in the feature space, as each model excels with different shaped feature spaces [59]. Similar modeling approaches have been used previously to assess fall risk, as the fall risk of non-fallers is considered low and fallers high [12,23]. Model hyperparameters were optimized with MATLAB’s Optimize Hyperparameters feature, with no access to test data, for each input feature set to provide the highest classification performance in terms of AUC.

### Deep learning fall risk classification

Based on previous literature [12], we also developed deep learning models for classifying walking fall risk. As used previously, we leveraged Long Short-Term Memory (LSTM) networks for this analysis. In our prior work, we demonstrated that the best classification performance was achieved considering four strides of data per input to the model, and showed that model performance changed with the number of strides considered [12]. For our analysis, we first optimized our networks to provide the best performance using four strides per input. This was done by extracting every walking bout with four or more strides and concatenating every consecutive four strides into a model input. These inputs contain three channels of raw acceleration from both the thigh and chest sensor from sequential strides. These data were arranged as a 6xN cell array, where the six represents the number of acceleration channels from both sensors and N represents the lengths of each stride summed. In the example case of a four-stride input, each input consisted of the thigh and chest acceleration from extracted stride 1 concatenated with the data from stride 2, then 3 and 4. Model outputs were a decision score for each input representing the posterior probability that the input belonged to a given class. Models were trained using LOSOCV, where n = 36 for training, n = 1 for validation, and n = 1 for testing for each training iteration (n = 35). A modified LOSOCV procedure was used for the deep learning methods to include an additional validation set to investigate the impacts of adjusting the number of training epochs; note, this method ensures that all data from a given subject is only included in one of the training, validation, or test sets. Using four stride inputs, we optimized our model over the number of LSTM or Bidirectional LSTM (BiLSTM) layers, training epochs, and number of hidden units based on the validation performance. The best two models were then selected and used to train inputs with one through twenty-two strides. The model referred to as LSTM 2 consisted of the following layers: an LSTM layer with 290 hidden units, 30% dropout, BiLSTM layer with 10 hidden units, 40% dropout, a fully connected layer, and softmax. The model referred to as LSTM 3 consisted of the following layers: an LSTM layer with 85 hidden units, 55% dropout, an LSTM layer with 85 hidden units, 55% dropout, an LSTM layer with 235 hidden units, 45% dropout, a fully connected layer, and softmax. The models were trained for 55 and 125 epochs, respectively, and both utilized adam optimization. Model denoted as ABC contained the subjects’ ABC score in the model inputs. Performance was assessed using area under the receiver operator curve (AUC) from the held-out test set for individual input predictions and for an aggregated model performance using the median classification from each subject.

## Results

A total of 15,097 free-living walking bouts were analyzed, with 9,135 (61%) identified as short, 4,840 (32%) as medium, and only 1,122 (7%) as long. Gait parameters differed considerably between bout lengths (Table 3). Notably, stride time CV, swing time, duty factor CV, RMS AP, and Freqd ML were significantly different between all bout durations. Stride time CV and RMS AP increased, and Freqd ML decreased with increasing duration. The increase in stride time CV at home may indicate greater stride to stride variability. Swing time of short and medium bouts was similar and greater than that observed during long bouts. Collectively, the increase in motion in the direction of travel and decrease in lateral motion implies that PwMS walk with greater stability during longer walking bouts.

**Table 3:** Difference of Medians Testing for free-living Gait parameters from differing bout lengths.

Comparison	Feature	Median 1	Median 2	p-value
4–8 vs. 12–28	Stride Time	1.16	1.16	0.460
	Stride Time CV	0.067	0.085	***< 0*.*001***
	Stance Time	0.71	0.71	0.644
	Swing Time	0.44	0.45	***0*.*012***
	Duty Factor	0.62	0.62	***0*.*025***
	Duty Factor CV	0.042	0.054	***< 0*.*001***
	RMS AP	0.14	0.14	***< 0*.*001***
	Freqd ML	0.62	0.60	***< 0*.*001***
4–8 vs. 32+	Stride Time	1.16	1.12	***< 0*.*001***
	Stride Time CV	0.067	0.079	***< 0*.*001***
	Stance Time	0.71	0.69	***< 0*.*001***
	Swing Time	0.44	0.42	***< 0*.*001***
	Duty Factor	0.62	0.62	***0*.*005***
	Duty Factor CV	0.04	0.051	***< 0*.*001***
	RMS AP	0.14	0.15	***< 0*.*001***
	Freqd ML	0.62	0.55	***< 0*.*001***
12–28 vs. 32+	Stride Time	1.16	1.12	***< 0*.*001***
	Stride Time CV	0.085	0.079	***< 0*.*001***
	Stance Time	0.71	0.69	***< 0*.*001***
	Swing Time	0.45	0.42	***< 0*.*001***
	Duty Factor	0.62	0.62	0.081
	Duty Factor CV	0.054	0.051	***0*.*011***
	RMS AP	0.14	0.15	***< 0*.*001***
	Freqd ML	0.61	0.55	***< 0*.*001***

Rank sum test with level of significance *α* = 0.05, significant results bolded and italicized. Stride, swing, and stance time in seconds; Freqd ML in Hz; RMS AP in √g; Duty Factor is unitless.

Significant differences between home and lab walking were found for all bout durations (Table 4). Freqd ML was significantly higher in free-living than in-lab conditions for all walking durations, with the shorter durations showing the largest differences. Stride time was also increased in free-living gait, with significant differences found in short, medium, and combined walking durations. As expected, these results imply that longer free-living walking bouts are the most similar to those completed in the lab, however, significant differences in the longer bouts remain. Specifically, the long free-living bouts have significantly higher entropy ratios, and Lyapunov exponents in the AP direction than those completed in the lab–each of which indicates a decrease in stability in free-living situations.

**Table 4:** Difference of Medians Testing for free-living and in lab gait parameters from differing bout lengths.

Comparison	Feature	Median In-lab	Median Free-living	p-value
Lab data vs 4–8	Stride Time	1.11	1.16	***< 0*.*001***
	Stride Time CV	0.06	0.067	***0*.*030***
	Stance Time	0.69	0.71	***0*.*001***
	Swing Time	0.42	0.44	***0*.*045***
	Duty Factor	0.62	0.62	0.473
	Duty Factor CV	0.045	0.042	***< 0*.*001***
	RMS AP	0.14	0.14	0.641
	Freqd ML	0.55	0.62	***< 0*.*001***
Lab data vs. 12–28	Stride Time	1.11	1.16	***< 0*.*001***
	Stride Time CV	0.06	0.085	***< 0*.*001***
	Stance Time	0.69	0.71	***0*.*001***
	Swing Time	0.42	0.45	**0.014**
	Duty Factor	0.62	0.62	0.979
	Duty Factor CV	0.045	0.054	***0*.*050***
	RMS AP	0.14	0.14	0.538
	Freqd ML	0.55	0.60	***< 0*.*001***
Lab data vs. 32+	Stride Time	1.11	1.12	0.160
	Stride Time CV	0.060	0.079	***< 0*.*001***
	Stance Time	0.69	0.69	0.206
	Swing Time	0.42	0.42	0.312
	Duty Factor	0.62	0.62	0.776
	Duty Factor CV	0.045	0.051	***0*.*050***
	RMS AP	0.14	0.15	***0*.*042***
	Freqd ML	0.55	0.55	0.205
	Lyapunov AP	0.006	0.014	***< 0*.*001***
	Lyapunov ML	0.013	0.012	0.194
	Entropy Ratio	1.78	2.55	***< 0*.*001***
Lab data vs All home data	Stride Time	1.11	1.16	***< 0*.*001***
	Stride Time CV	0.060	0.064	0.821
	Stance Time	0.69	0.71	***0*.*002***
	Swing Time	0.42	0.44	***0*.*037***
	Duty Factor	0.62	0.62	0.673
	Duty Factor CV	0.045	0.042	0.195
	RMS AP	0.14	0.14	0.948
	Freqd ML	0.55	0.61	***< 0*.*001***

Unit of stride, swing, and stance duration is seconds. Unit Freqd ML is Hz, RMS AP is √g, and Duty Factor is unitless. All p-values found using a rank sum test using a significance threshold of 0.05, significant results are bolded and italicized.

**Fig 2:**
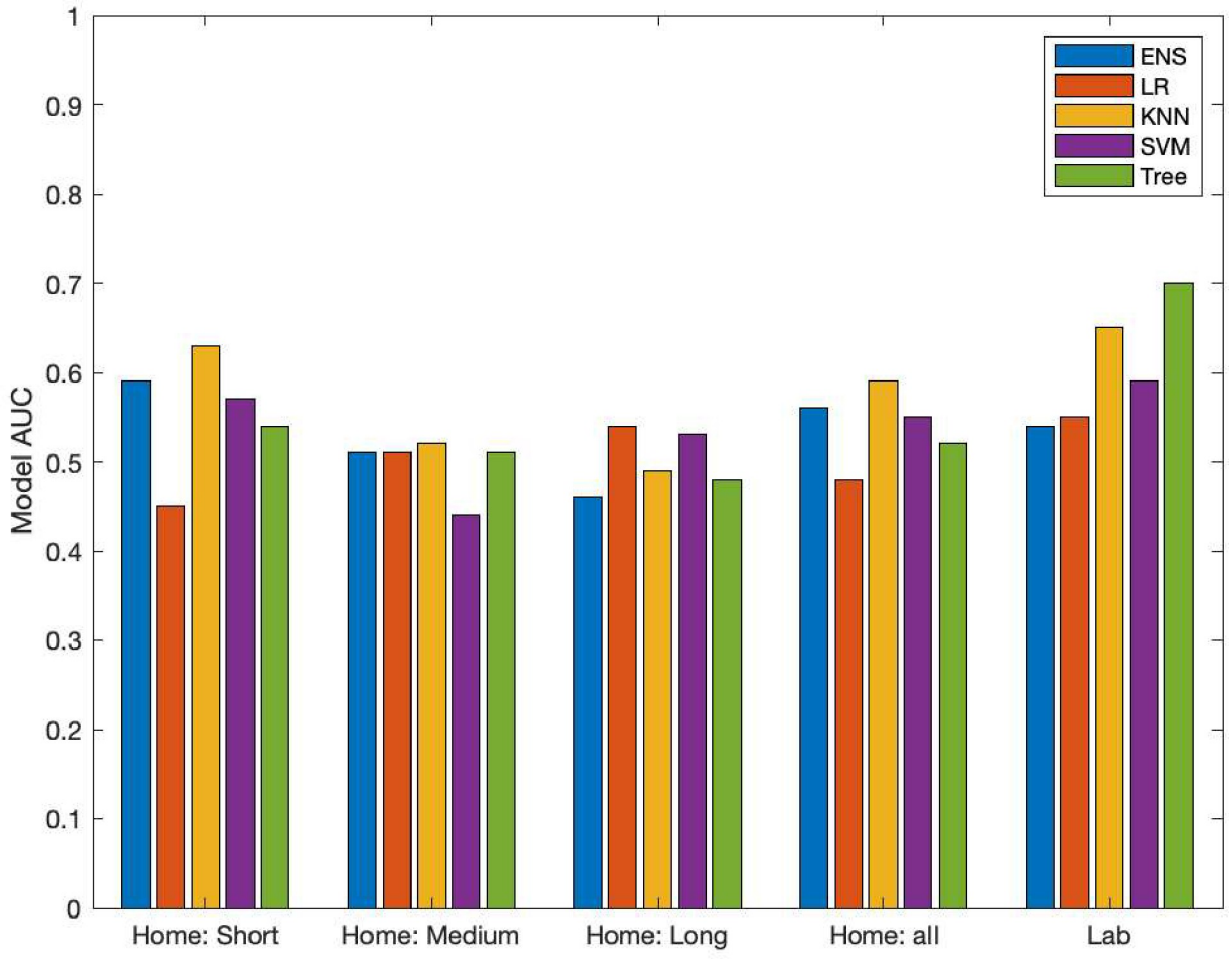
Fall Risk Classification Model AUC for Short Home, Medium Home, Long Home, All Home, and In-Lab Walking Bouts.

Significant differences between the gait parameters of fallers and non-fallers were observed for short and long walking bouts as seen in Table 5. Notably, in short walking bouts, we see fallers have a lower RMS AP, signifying higher impairment as expected [49]. This suggests short and long walking bouts are more sensitive to fall risk compared to medium duration walking bouts. Fall classification models trained on the gait parameters explored in this study performed best on lab walking bouts and short walking bouts when considering home walking only (see AUC of knn for 8-seconds or less in Fig 2).

**Table 5:** Significant Differences of medians of gait parameters for fallers vs non-fallers from differing bout lengths.

Bout Duration	Feature	Median Faller	Median Non-faller	p-value
4–8	Max RMS AP	0.25	0.36	***0*.*012***
	Med RMS AP	0.13	0.15	***0*.*019***
	Mean RMS AP	0.14	0.15	***0*.*019***
	95^th^ P RMS AP	0.20	0.22	***0*.*037***
12–28	No significant differences			
32+	5^th^ P Duty Factor	0.57	0.58	***0*.*017***
	Mean Duty Factor	0.61	0.62	***0*.*017***
	Med Duty Factor	0.61	0.63	***0*.*018***
	Max Duty Factor	0.65	0.66	***0*.*029***
	Max Stance Time	0.79	0.86	***0*.*033***
	95^th^ P Duty Factor	0.64	0.65	***0*.*038***
	Min Entropy Ratio	1.06	0.92	***0*.*039***
	Med Swing Time	0.43	0.43	***0*.*040***

Unit of step, swing, and stance duration is seconds. Unit Freqd ML is Hz, RMS AP is √g, and Duty Factor is unitless. All p-values found using a rank sum test using a significance threshold of 0.05, significant results are bolded and italicized. 5^th^ or 95^th^ P: 5^th^ or 95^th^ percentile; Med: Median.

**Fig 3:**
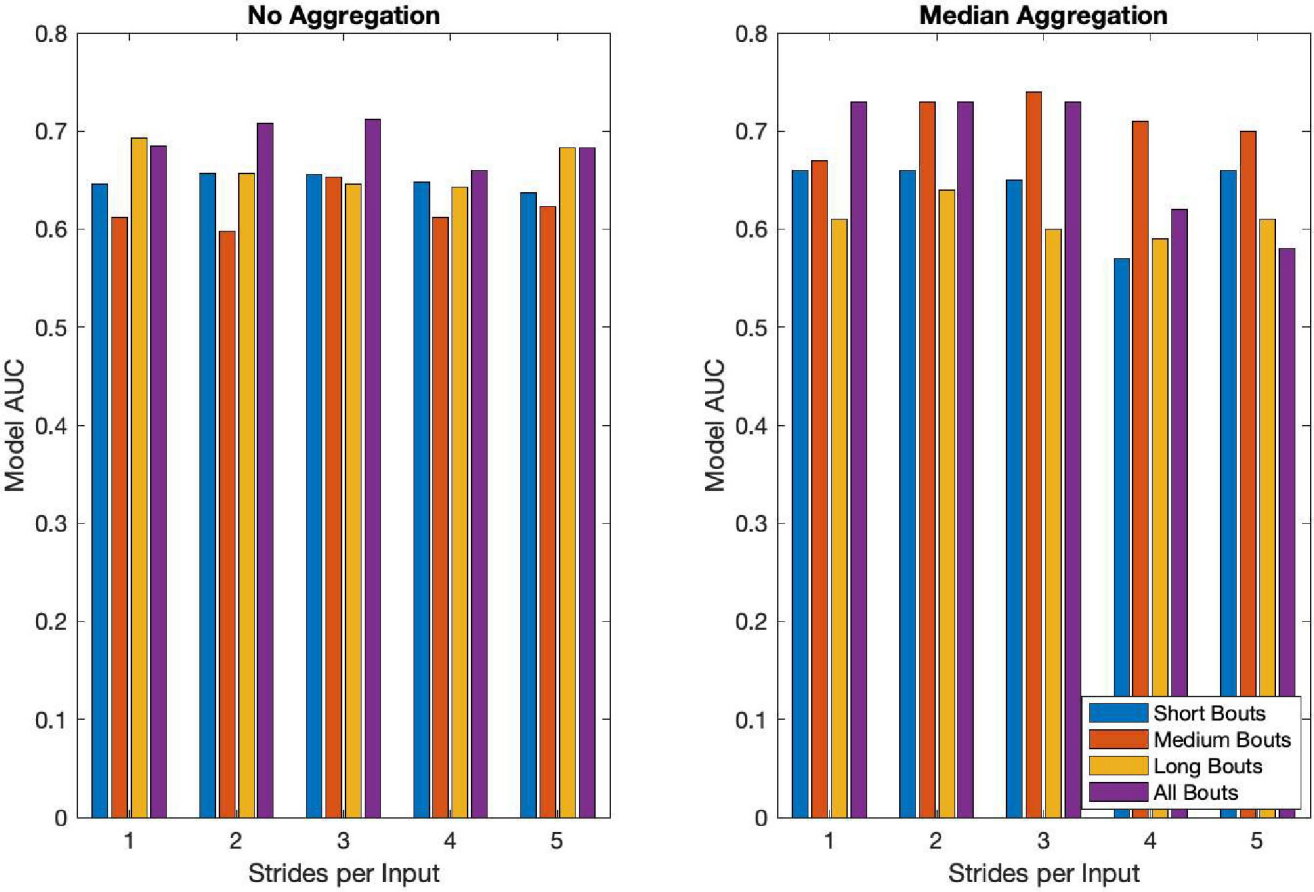
Fall risk classification model AUC for best performing deep learning model from short, medium, long, and all walking bouts for 1–5 inputs per stride without aggregation (left) and with median aggregation (right).

**Fig 4:**
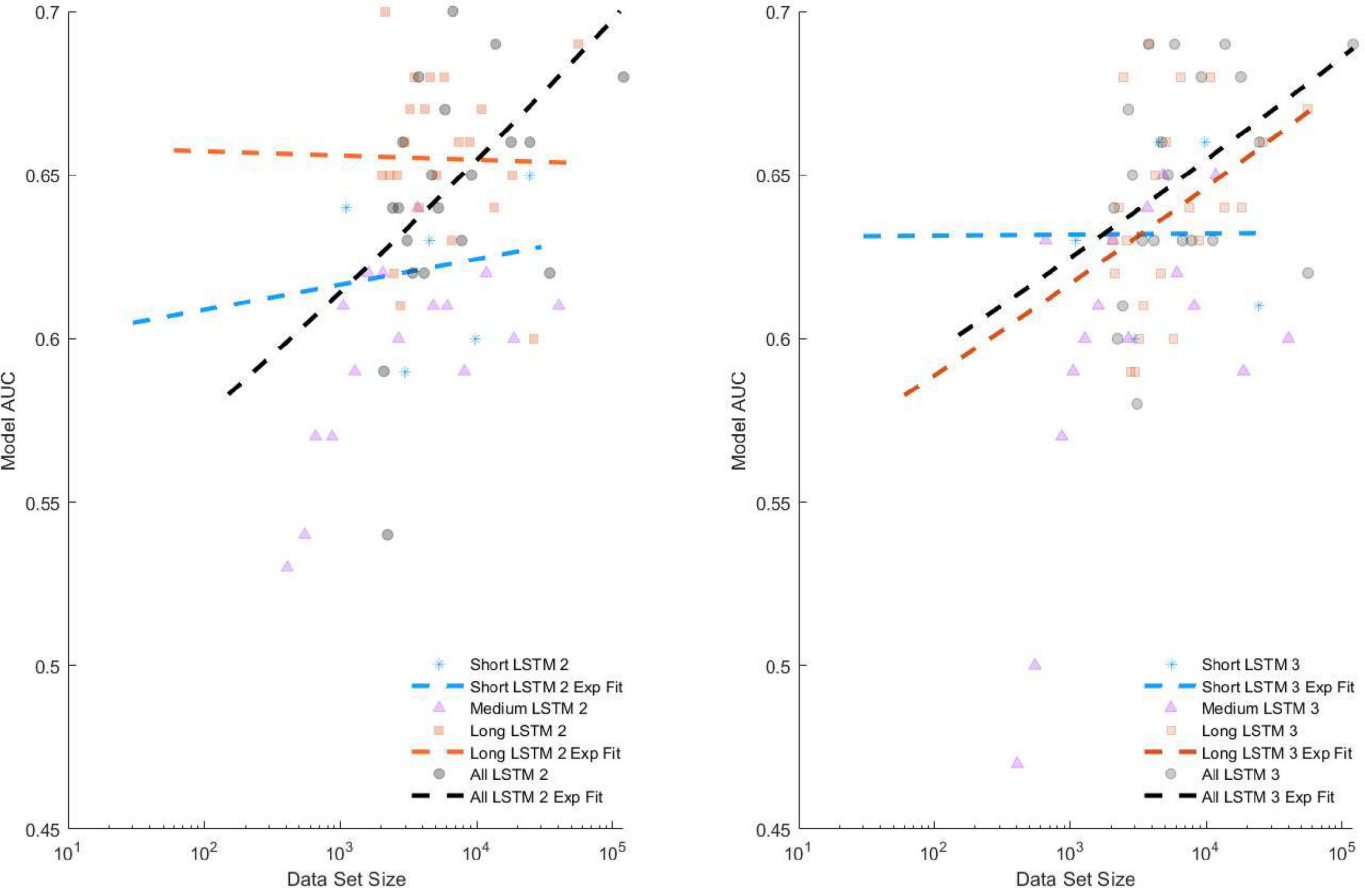
Fall risk classification model AUC for LSTM 2 ABC and LSTM 3 ABC for all stride durations colored by bout length, short (blue), medium (pink), long (red), and all (black), plotted against the training set size for each model showing increasing performance, increasing exponential fits, for several model/bout configurations with data set size. Notice the stronger increasing trends in the right LSTM 3 plots in all and long bouts compared to the LSTM 2 plot. Additionally notice the increase in slope of short LSTM 2 compared to short LSTM 3. This suggests that the larger models are needed to capture variability in longer bouts and smaller models perform better with shorted bouts. Note, the medium trend (not shown) was strongly increasing for both LSTM 2 and 3.

The best overall feature-based fall classifier was a decision tree model using lab walking bouts. Performance of this model was characterized by an AUC of 0.70. The best performing feature-based home fall classification model was a KNN with short bout inputs achieving an AUC of 0.63. The KNN model also performed best for medium walking bouts, and all home walking bouts, providing AUCs of 0.52 and 0.59 respectively. The best performing feature-based model on long home walking bouts was the LR model, with an AUC of 0.54. The best performing deep learning model was the LSTM 2 trained on inputs with 22 strides with ABC for all walking bouts using the median aggregation with an AUC of 0.76. The best performing non-aggregated model was LSTM 3 with ABC trained on input with three strides from all walking bouts. Detailed performance of the models can be found in S1 Table, located in the appendix. Fig 3 reveals that when using the median aggregation, the performance of the medium bouts sees a notable improvement compared to the other bout lengths, suggesting that the aggregation may be reducing some of the noise inherent in that walking duration. Fig 4 shows the performance of each model relative to its input size, which seems to show that short, medium, and long bouts continue to increase their performance with dataset size. In contrast, the all-bouts models seem to achieve stable performance levels as dataset size is increased.

The impact of these results is twofold. First, considering the feature-based methods, these models show that overall fall risk is best predicted by lab walking and that for free living gait fall risk is best predicted by considering short-duration walking bouts. Second, we show that deep learning models trained on raw stride data perform better on home data when considering all bouts and using a larger number of strides per input. As the strides per input increase, the gait is likely more similar to steady-lab walking than variable free-living walking. With this hypothesis, both the feature-based models and deep learning modeling reach a similar conclusion (supported by Table 4), namely that many consecutive clean strides are needed to classify fall risk using this framework. Fig 4, however, shows that the performance of both models using medium, and all bouts seems to increase with dataset size. Short bouts using the LSTM 2 model also appear to show an increasing performance with more data, however, the limited range of data set sizes for small data limits the ability to find trends. Performance using long bouts is better captured using a larger model such as LSTM 3 which shows improvement with increasing data set size compared to the smaller LSTM 2 model where this trend does not exist. These trends, however, suggest that the addition of more data, and perhaps models that can better account for the variability may provide better performance.

## Discussion

In this paper we present a novel wearable sensor dataset collected from PwMS. This dataset includes data from a supervised laboratory visit, neurologist assessments, patient reported measures, and an unsupervised monitoring period for each PwMS. Novel findings from the in-lab period of this study have found walking and 30-second chair stand tests to be indicative of fall risk [12,40]. Analysis of free-living 30-second chair stand tests and posture transitions have also revealed relationships with fall risk and impairment [41]. Herein, we presented a preliminary analysis of walking in the free-living environment as it relates to fall risk and differing lengths of walking bouts.

The main finding from this study is that both gait bout length and environment influence wearables-based fall classification in PwMS. Specifically, the best performance overall was observed for classifiers that use lab data or long, steady walking bouts that are similar to the lab (Fig 2 and S1 Table). The best performing feature-based model on free-living data was trained on short walking bouts, suggesting that short free-living bouts may be worth further exploration with a more nuanced feature-set. Our best un-aggregated deep learning model was trained on 3-stride inputs from all bouts. We hypothesize this performed best because deep learning models require a large amount of data to train and considering all bouts allows the model access to far more data than just the short bouts.

Compared to other fall risk classification studies, the performance of our remote fall risk classifier is on par with many lab-based studies, but still lags behind the best approaches. In-lab studies have achieved AUCs between 0.73 and 0.79 in older adults [60]. In PwMS an in-lab study using the dynamic gait index achieved an AUC of 0.80 [61] and our prior work, where a deep learning model was used on walking data, achieved an AUC of 0.88 [12]. The difference between our previous lab-based fall risk performance of 0.88 and the performances presented herein highlights a key challenge in using deep learning methods on remote data. Namely, that the model must be able to reconcile the additional variability in gait observed under free living conditions. Performance was observed to increase with increasing dataset size in Fig 4, indicating that deep learning approaches may be able to learn appropriate representations of the data to account for this variability, but the dataset considered here is likely not large enough. By open-sourcing these data, we aim to allow future researchers to realize the promise of deep learning for fall risk classification in PwMS.

Our finding that bout length and environment influence discrimination of fallers from non-fallers is in agreement with similar gait-based classification applications in patients with neurological disorders. For example, one study found that the features that best discriminate between PwMS and healthy controls were different when using lab data and home data [62]. Similarly, other studies demonstrate that shorter walking bouts provide better discriminative power when trying to identify a person with Parkinson’s Disease versus healthy controls as well [54], and pace is different in free-living walking compared to in-lab for PwMS [24].

The influence of bout length and environment on fall classification is likely related to the observed differences in the various gait descriptors used as features in the classification models (Tables 3 and 4). This finding contributes more generally to the growing body of evidence that controlled in-lab observations of gait are not representative of free-living conditions. In the current study, this discrepancy was more pronounced for short and medium walking bouts than for long; a finding which is likely due to the fact that the in-lab walking bout was, by our definition, a long walking bout (one-minute long). Differences observed between gait parameters calculated at differing bout lengths (see Table 3) show that stride, stance, and swing time decrease as bout duration increases. This likely means that PwMS are increasing their cadence for longer walking bouts. The observed decrease in ML frequency dispersion with increasing bout length also suggests PwMS walk more steadily, with less lateral motion for long duration walking bouts. These results are consistent with Storm et al., who found that gait pace significantly increased and variability significantly decreased with increasing bout length [24]. Karle et al. found little correlation between an in-lab 2-minute walk test and free-living walking [25]. In older adults, Najafi et. al observed significantly different walking strategies between short and long walks [30]. The reason for this change in gait is unknown, however, it can be speculated that shorter walking bouts may elicit more goal-direction actions towards activities other than walking while longer bouts are more purposeful [54]. Further expanding on the involuntary nature of shorter walking bouts, subjects may be more likely to be dual-task walking, in other words focused on more than just walking, and may be more impacted by the start-up and stopping strides [63]. This conjecture aligns with research on dual-task walking in PwMS that shows dual-task walking is more discriminative of impairment than single task walking [64].

The distribution of bout length in free-living gait from the current sample (61% short, 32% medium, 7% long) is comparable to what has been observed in Parkinson’s disease [54]. Preliminarily, this consistency across populations may suggest a phenomenon that is representative of free-living gait more generally. This raises important questions concerning remote gait analysis more broadly to be investigated in future research. For example, does bout length explain the free-living vs. in-lab discrepancy in various gait descriptors consistently observed across multiple populations? If the observed distribution of bout lengths does generalize, then free-living gait is generally short-bout and less purposeful while long, purposeful walking is rare. Further, given that in-lab investigations of gait are controlled and supervised by a clinician or researcher, they may naturally elicit more purposeful walking from the subject (even over short distances) and be less prone to the impacts of fatigue inherent in daily-life. Thus, differences in free-living and in-lab gait may be explained by the fact that aggregated metrics of free-living data (e.g., average gait speed in a 24-hour period) are dominated by those characteristic of short-duration gait bouts (> 50%) and is influenced to a far lesser extent by metrics characteristic of long-duration and purposeful gait bouts (< 10%).

There are several limitations to our study. First, our relatively small sample with moderate to low impairment may not generalize to a larger population of PwMS, particularly PwMS with EDSS greater than six, who were not represented in this study. Other studies utilize different sensing modalities that provide gait speed, which was not available with our data collection set up. Additionally, our analysis methods require a four second window to be classified as non-walking to denote separate bouts. This definition of what defines a separate bout may impact certain gait quantity metrics, however, our study uses gait quality metrics which have been shown to be independent of temporal gait bout definitions [65]. Lastly, symptoms in PwMS are known to fluctuate over differing time scales and thus, 48 hours may not have been a long enough collection time to provide an accurate depiction of each participant’s overall mobility status [9]. Future work will be needed to determine how gait parameters vary in PwMS on longer time scales.

With the presented dataset, we hope to alleviate one of the most challenging issues related to human subject research with wearables: not having enough data. Publicly available datasets gathered from PwMS are largely related to medical imaging [66–68] and medication [69]. One dataset tackles a related issue: remote fall detection in PwMS [70], however, it is lacking data from PwMS who have yet to become recurrent fallers, preventing the investigation of gait as it relates to distinguishing fallers from non-fallers and potentially fall-risk prediction. Utilizing the presented data, potentially with other collected or open-source data, researchers may be able to leverage deep learning to enhance the performance of their digital biomarkers and phenotypes, and particularly for detecting fall risk in PwMS in both lab and free-living environments. With that said, the vision of real-time fall risk monitoring comes with challenges such as when and how to alert the user to an elevated fall risk, how or if to integrate with their comprehensive care, and these data need to be protected. These are all challenges that will need to be addressed and researched in the future as we move towards a preventative care paradigm for falls in PwMS and other populations with balance and mobility impairment.

## Conclusion

Herein, we introduce a new open-source dataset featuring activities of daily living and functional assessments from a lab environment as well as two days of free-living data in PwMS. This dataset features data from PwMS with lower impairment, including approximately half that do not yet have recurrent fall histories. As an example use case, we present a study of gait in the free-living environment. In this study, we explored differences in gait parameters calculated on short, medium, and long duration walking bouts. Specifically, we investigated the significant differences between durations of home walking and in-lab walking and fall classification performance using features calculated from differing walking durations. Several significant differences were found between the gait parameters at differing durations. We also demonstrated that fall risk classification performance using gait changes based on walking bout duration. Short walking bouts, 8 seconds or less, were found to be the most discriminative, providing significant differences between fallers and non-fallers and providing the best free-living fall risk classification performance in the feature-based models. Additionally, we demonstrated that in-lab walking gait parameters are significantly different from free-living walking, at all durations, and that fall risk models used on remote data should be trained with remote data. While future studies are required to assess the reliability of these findings over a longer time period, these results suggest that remote gait analysis may benefit from focusing on short walking bouts in future analysis.

## Supporting information

S1 Table Performance of deep learning models by number of strides and data considered. LSTM: Long-Short Term Memory Neural Network; LSTM 2: Model with one LSTM layer and one BilSTM layer; LSTM 3: Model with LSTM Layers; AGG: Aggregation technique (none or median of all remote stride observations); AUC: Area Under the Receiver Operating Characteristic Curve; ABC: Activity Specific Balance Confidence added as input feature; N/A: Not enough data available to extract specified number of strides from each subject.
(DOCX)
