# Supplementary material for: Open-source dataset reveals relationship between walking bout duration and fall risk classification performance in persons with multiple sclerosis
Source: PLOS Digit Health. 2022 Oct 18;1(10):e0000120. doi: 10.1371/journal.pdig.0000120 (PMC9931255; doi:10.1371/journal.pdig.0000120)
Supplement: S1 Table — LSTM: Long-Short Term Memory Neural Network; LSTM 2: Model with one LSTM layer and one BilSTM layer; LSTM 3: Model with LSTM Layers; AGG: Aggregation technique (none or median of all remote stride observations); AUC: Area Under the Receiver Operating Characteristic Curve; ABC: Activity Specific Balance Confidence added as input feature; N/A: Not enough data available to extract specified number of strides from each subject. (DOCX) [file pdig.0000120.s001.docx]

S1 TABLE

performance of deep learning models by number of strides and amount of data considered

| Strides | AGG | Short Model | Short AUC | Medium Model | Medium AUC | Long Model | Long AUC | All Model | All AUC |
| --- | --- | --- | --- | --- | --- | --- | --- | --- | --- |
| 22 | None | N/A | N/A | N/A | N/A | LSTM 2 ABC | 0.68 | LSTM 3 ABC | 0.64 |
|  | Median | N/A | N/A | N/A | N/A | LSTM 3 ABC | 0.69 | LSTM 2 ABC | ***0.76*** |
| 21 | None | N/A | N/A | N/A | N/A | LSTM 2 ABC | 0.70 | LSTM 3 ABC | 0.60 |
|  | Median | N/A | N/A | N/A | N/A | LSTM 2 ABC | 0.68 | LSTM 3 ABC | 0.69 |
| 20 | None | N/A | N/A | N/A | N/A | LSTM 2 ABC | 0.65 | LSTM 2 ABC | 0.64 |
|  | Median | N/A | N/A | N/A | N/A | LSTM 3 ABC | 0.67 | LSTM 2 ABC | 0.67 |
| 19 | None | N/A | N/A | N/A | N/A | LSTM 3 ABC | 0.68 | LSTM 3 ABC | 0.67 |
|  | Median | N/A | N/A | N/A | N/A | LSTM 3 ABC | 0.64 | LSTM 2 ABC | 0.63 |
| 18 | None | N/A | N/A | N/A | N/A | LSTM 2 ABC | 0.65 | LSTM 2 ABC | 0.66 |
|  | Median | N/A | N/A | N/A | N/A | LSTM 2 ABC | 0.68 | LSTM 3 ABC | 0.64 |
| 17 | None | N/A | N/A | N/A | N/A | LSTM 2 ABC | 0.61 | LSTM 2 ABC | 0.63 |
|  | Median | N/A | N/A | N/A | N/A | LSTM 3 ABC | 0.62 | LSTM 2 ABC | 0.62 |
| 16 | None | N/A | N/A | LSTM 2 | 0.59 | LSTM 2 ABC | 0.66 | LSTM 3 ABC | 0.63 |
|  | Median | N/A | N/A | LSTM 2 ABC | 0.61 | LSTM 2 ABC | 0.64 | LSTM 2 ABC | 0.64 |
| 15 | None | N/A | N/A | LSTM 3 | 0.60 | LSTM 2 ABC | 0.67 | LSTM 3 ABC | 0.69 |
|  | Median | N/A | N/A | LSTM 3 ABC | 0.63 | LSTM 2 ABC | 0.66 | LSTM 3 ABC | 0.72 |
| 14 | None | N/A | N/A | LSTM 3 ABC | 0.63 | LSTM 2 ABC | 0.68 | LSTM 3 ABC | 0.63 |
|  | Median | N/A | N/A | LSTM 2 ABC | 0.64 | LSTM 2 ABC | 0.69 | LSTM 2 ABC | 0.65 |
| 13 | None | N/A | N/A | LSTM 3 ABC | 0.57 | LSTM 3 ABC | 0.69 | LSTM 3 ABC | 0.66 |
|  | Median | N/A | N/A | LSTM 3 ABC | 0.62 | LSTM 3 ABC | 0.67 | LSTM 2 ABC | 0.69 |
| 12 | None | N/A | N/A | LSTM 2 ABC | 0.61 | LSTM 2 ABC | 0.67 | LSTM 3 ABC | 0.65 |
|  | Median | N/A | N/A | LSTM 3 ABC | 0.66 | LSTM 2 ABC | 0.69 | LSTM 2 ABC | 0.69 |
| 11 | None | N/A | N/A | LSTM 3 ABC | 0.60 | LSTM 2 ABC | 0.68 | LSTM 3 ABC | 0.69 |
|  | Median | N/A | N/A | LSTM 2 ABC | 0.66 | LSTM 2 ABC | 0.64 | LSTM 3 ABC | 0.68 |
| 10 | None | N/A | N/A | LSTM 2 ABC | 0.62 | LSTM 3 ABC | 0.66 | LSTM 2 ABC | 0.70 |
|  | Median | N/A | N/A | LSTM 2 ABC | 0.68 | LSTM 3 ABC | 0.63 | LSTM 2 ABC | 0.74 |
| 9 | None | N/A | N/A | LSTM 3 ABC | 0.63 | LSTM 2 ABC | 0.68 | LSTM 2 ABC | 0.62 |
|  | Median | N/A | N/A | LSTM 2 ABC | 0.67 | LSTM 2 ABC | 0.63 | LSTM 2 ABC | 0.64 |
| 8 | None | N/A | N/A | LSTM 2 ABC | 0.64 | LSTM 3 ABC | 0.68 | LSTM 3 ABC | 0.68 |
|  | Median | N/A | N/A | LSTM 2 ABC | 0.71 | LSTM 3 ABC | 0.69 | LSTM 3 ABC | 0.59 |
| 7 | None | N/A | N/A | LSTM 3 ABC | 0.64 | LSTM 2 ABC | 0.66 | LSTM 2 ABC | ***0.71*** |
|  | Median | N/A | N/A | LSTM 2 ABC | 0.65 | LSTM 2 ABC | 0.63 | LSTM 2 ABC | 0.63 |
| 6 | None | N/A | N/A | LSTM 3 ABC | 0.65 | LSTM 2 ABC | 0.66 | LSTM 2 ABC | 0.69 |
|  | Median | N/A | N/A | LSTM 3 ABC | 0.75 | LSTM 2 ABC | 0.64 | LSTM 2 ABC | 0.64 |
| 5 | None | LSTM 2 ABC | 0.64 | LSTM 3 ABC | 0.62 | LSTM 3 ABC | 0.68 | LSTM 3 ABC | 0.68 |
|  | Median | LSTM 3 ABC | 0.66 | LSTM 2 ABC | 0.70 | LSTM 3 ABC | 0.61 | LSTM 2 ABC | 0.58 |
| 4 | None | LSTM 3 ABC | 0.60 | LSTM 2 ABC | 0.61 | LSTM 2 ABC | 0.64 | LSTM 2 ABC | 0.66 |
|  | Median | LSTM 3 ABC | 0.57 | LSTM 3 ABC | 0.71 | LSTM 2 ABC | 0.59 | LSTM 2 ABC | 0.62 |
| 3 | None | LSTM 3 ABC | 0.66 | LSTM 3 ABC | 0.65 | LSTM 2 ABC | 0.65 | LSTM 3 ABC | ***0.71*** |
|  | Median | LSTM 3 ABC | 0.65 | LSTM 3 ABC | 0.74 | LSTM 3 ABC | 0.60 | LSTM 3 ABC | 0.73 |
| 2 | None | LSTM 3 ABC | 0.66 | LSTM 2 ABC | 0.60 | LSTM 3 ABC | 0.66 | LSTM 2 ABC | ***0.71*** |
|  | Median | LSTM 3 ABC | 0.66 | LSTM 2 ABC | 0.73 | LSTM 3 ABC | 0.64 | LSTM 2 ABC | 0.73 |
| 1 | None | LSTM 2 ABC | 0.65 | LSTM 2 ABC | 0.61 | LSTM 2 ABC | 0.69 | LSTM 3 ABC | 0.69 |
|  | Median | LSTM 2 ABC | 0.66 | LSTM 3 ABC | 0.67 | LSTM 2 ABC | 0.61 | LSMT 3 ABC | 0.73 |

LSTM: Long-Short Term Memory Neural Network; LSTM 2: Model with one LSTM layer and one BilSTM layer; LSTM 3: Model with LSTM Layers; AGG: Aggregation technique (none or median of all remote stride observations); AUC: Area Under the Receiver Operating Characteristic Curve; ABC: Activity Specific Balance Confidence added as input feature; N/A: Not enough data available to extract specified number of strides from each subject.
